# Atlanta’s Milk Supply

**Published:** 1903-09

**Authors:** 


### ATLANTA'S MILK SUPPLY.

THE recent investigation of the milk supply of the city of Atlanta, by Dr. R. W. Hynds, assistant city chemist and bacteriologist, brought out some facts in connection with the cleanliness of the specimens that are liable to discourage the public's appetite for this nutrient fluid for quite a while. With but few exceptions the samples were shown to be dirty, very dirty, or filthy. The report was scarcely a surprise to the citizens of the city, as they had become so accustomed to hooking stable manure out of their milk that without it they would have felt a sense of strangeness. Habituation had drowned out criticism. They did not seem to worry much over the difference between excretions and secretions. But when it was made known that the familiar sediment contained pus, blood, flies and the remains of other dipterous insects that did not properly belong there, there was some disposition to turn from the bland juice of the cow to the stronger extractions of grape and grain. In his rejoinder to Dr. Hynd's report, the representative of the dairymen sought, by picking flaws in the document, to throw discredit upon the entire report. But it did not need the eye of an expert to see the floating and precipitated filth, so that the question of the relative amount of butter fats is scarcely pertinent, nor would the accuracy or inaccuracy of the report in this regard disturb the main contention that the milk was dirty. Fine-spun argument may gracefully drape itself over commonplace facts, like bedewed cobwebs over horse droppings, but they fail to conceal the nature of the substance beneath.

However shrill the denunciation of the milk men may be against the report, their product has been much cleaner since it appeared; and although they virtuously asseverate the virgin purity of the goods supplied, there has been some gilding of refined gold and some perfume added to the violet since the clear beam of science illuminated the interior of their cans and bottles.

